# Nociception and Conditioned Fear in Rats: Strains Matter

**DOI:** 10.1371/journal.pone.0083339

**Published:** 2013-12-23

**Authors:** Manon W. H. Schaap, Hugo van Oostrom, Arie Doornenbal, José van 't Klooster, Annemarie M. Baars, Saskia S. Arndt, Ludo J. Hellebrekers

**Affiliations:** 1 Department of Clinical Sciences of Companion Animals, Faculty of Veterinary Medicine, Utrecht University, Utrecht, The Netherlands; 2 Rudolf Magnus Institute of Neuroscience, Utrecht, The Netherlands; 3 Department of Animals in Science & Society, Faculty of Veterinary Medicine, Utrecht University, Utrecht, The Netherlands; Université catholique de Louvain, Belgium

## Abstract

When using rats in pain research, strain-related differences in outcomes of tests for pain and nociception are acknowledged. However, very little is known about the specific characteristics of these strain differences. In this study four phylogenetically distant inbred rat strains, i.e. Wistar Kyoto (WKY), Fawn Hooded (FH), Brown Norway (BN) and Lewis (LE), were investigated in different tests related to pain and nociception. During Pavlovian fear conditioning, the LE and WKY showed a significantly longer duration of freezing behaviour than the FH and BN. Additionally, differences in c-Fos expression in subregions of the prefrontal cortex and amygdala between rat strains during retrieval and expression of conditioned fear were found. For example, the BN did not show recruitment of the basolateral amygdala, whereas the WKY, FH and LE did. During the hot plate test, the WKY and LE showed a lower thermal threshold compared to the BN and FH. In a follow-up experiment, the two most contrasting strains regarding behaviour during the hot plate test and Pavlovian fear conditioning (i.e. FH and WKY) were selected and the hot plate test, Von Frey test and somatosensory-evoked potential (SEP) were investigated. During the Von Frey test, the WKY showed a lower mechanical threshold compared to the FH. When measuring the SEP, the FH appeared to be less reactive to increasing stimulus intensities when considering both peak amplitudes and latencies. Altogether, the combined results indicate various differences between rat strains in Pavlovian fear conditioning, nociception related behaviours and nociceptive processing. These findings demonstrate the necessity of using multiple rat strains when using tests including noxious stimuli and suggest that the choice of rat strains should be considered. When selecting a strain for a particular study it should be considered how this strain behaves during the tests used in that study.

## Introduction

Inbred rat strains are becoming more and more valuable in pain research because of the availability of extensive genomic information and transgenic technologies in these species [1], enabling the study of genetic background and the role of individual proteins in pain. Although strain-related differences in outcomes of tests for pain and nociception are acknowledged in general terms [2], [3], the exact characteristics of inbred strain-related differences in read-out parameters of pain and nociception are not well established. Thermal and mechanical (anti)nociception in rats and mice are commonly studied using the hot plate and Von Frey test, respectively [4]. In the hot plate test, the animal is placed on a plate heated at a fixed temperature until a predetermined behavioural endpoint is observed, typically a hind-paw lick or jump response [5], [6]. In the automated Von Frey test, a probe is placed under the hind paw which gradually builds up force, until the animal withdraws the paw. Differences in response latencies (i.e. hot plate test) and withdrawal threshold (i.e. Von Frey test) between animals are considered to reflect differences in thermal and mechanical nociceptive threshold, respectively [4]. Although the literature documents the use of many different rat strains, very little is known about differences in behaviour expressed during the hot plate and Von Frey test.

The Pavlovian fear conditioning paradigm is often applied to assess the neural mechanisms and consequences of fear (often using noxious stimuli). Furthermore, this paradigm has also been used to correlate cortical processing of nociceptive stimuli and pain unpleasantness in rats [7]. In this paradigm, animals are trained to associate a neutral stimulus (conditioned stimulus (CS)) with an aversive (typically painful) stimulus (unconditioned stimulus (US)). After the training phase, presentations of the CS evoke aversive behavioural responses (conditioned response (CR), i.e. fear related behaviour). These responses serve as a measure for the adversity to the US and are considered to represent an expression of the pain experienced from US exposure [8]. Furthermore, the expression of CR has been shown to correlate with the cortical processing of the US (i.e. nociceptive stimuli) [7]. Although the exact neural substrates and their roles involved in expression of CR are not known, several brain areas are suggested to play a role, including the amygdala, hippocampus and prefrontal cortex [9]–[13]. Strain differences in Pavlovian fear conditioning are reported [14], [15], but only few strains have been compared directly. Furthermore, it is not known whether there are strain differences regarding recruitment of associated brain areas.

Cortical processing of nociceptive stimuli is studied with the somatosensory evoked potential (SEP), a time- and stimulus-locked fragment of the electroencephalogram. The SEP waveform is described by the latency (the time of occurrence) and amplitude (height) of peaks of interest, both of which can be analyzed in a highly standardized and objective way. The SEP represents the processing of noxious stimuli [16], correlates well to adversity to pain in animals [7], [17] and is altered by anaesthetic and analgesic treatment [18], [19]. Earlier studies showed that lower stimulus intensities and higher dosages of anaesthetics lead to longer latency times and lower amplitudes [18], [20], [21]. Therefore, the SEP can be considered a potential method to quantify acute pain. Currently, SEPs are used in animals to increase knowledge about pain [22]. However, strain related differences in the cortical processing of pain are not described in the literature to date.

Knowledge about specific characteristics of strain differences potentially facilitates the unravelling of fundamental mechanisms involved in pain and analgesia, such as identification of involved phenotypes (i.e. behavioural characteristics). To this end, phenotyping of pain-related phenomena with both behavioural and cerebral read-out parameters using thermal, mechanical and electrical stimuli in four phylogenetically distant inbred rat strains [23] was performed in this study.

## Materials and Methods

**Figure 1:**
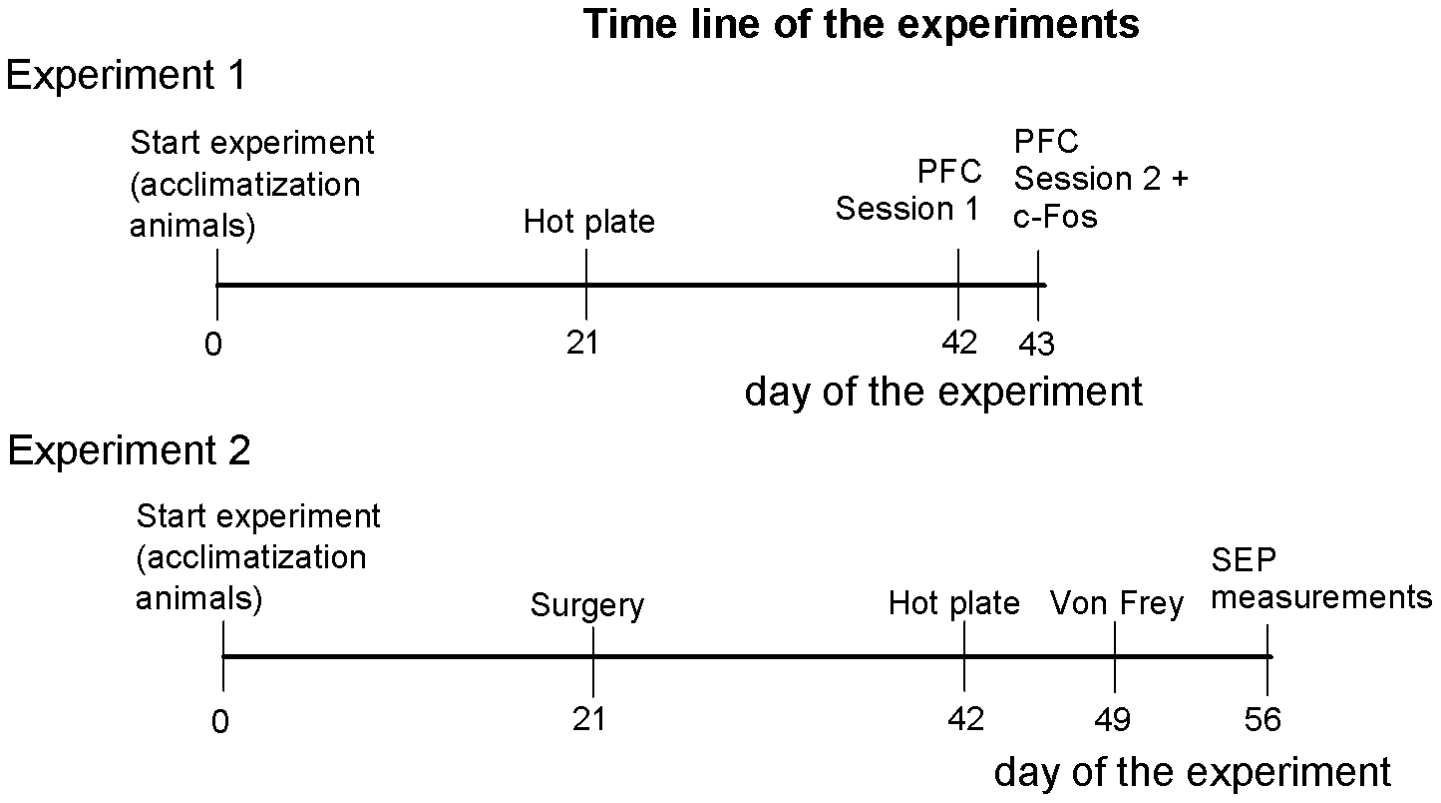
Time line of the study (in days). Two separate experiments were performed during this study. At the start of each experiment, naïve rats were used. Rats were allowed to adapt to our laboratory for 3 weeks, before experimental procedures started. PFC =  Pavlovian fear conditioning.

During this study, two separate experiments were performed. At the start of each experiment, naïve animals were used. Below, the materials and methods are described per experiment. In Fig. 1 the general time line of each experiment is depicted.

### Ethical note

The experimental protocols (DEC-DGK numbers: 2010.I.12.257 and 2011.I.08.079) were approved by the Animal Experiments Committee of the Academic Biomedical Centre, Utrecht, The Netherlands. The Animal Experiments Committee based its decision on ‘De Wet op de Dierproeven’ (the Dutch ‘Experiments on Animals Act’, 1996) and on the ‘Dierproevenbesluit’ (the Dutch ‘animal experiments decree’, 1996). Both documents are available online at http://wetten.overheid.nl. Further, all animal experiments followed the ‘Principles of Laboratory Animal Care’ and refer to the Guidelines for the Care and Use of Mammals in Neuroscience and Behavioural Research (National Research Council 2003).

### Experiment 1

#### Animals

Four different adult male inbred rat strains from a single source (Elevage Janvier, Le Genet St. Isle, France; n = 14 for each strain; 8 weeks old at the time of arrival), were used, including Wistar Kyoto (WKY, denomination: WKY/KyoRj), Fawn Hooded (FH, denomination: Rjlbm: FH), Brown Norway (BN, denomination: BN/OrlRj) and Lewis (LE, denomination: LE/OrlRj). Body weights (±SEM) were 225.07 (±2.02), 202.43 (±2.92), 163.64 (±1.13) and 233.57 (±2.04) gram at the time of arrival, respectively.

#### Housing conditions

All rats were housed individually in clear 1500U Eurostandard Type IV S cages of 48×37.5×21 cm (Techniplast, Buguggiate, Italy). Rats were provided with bedding material (Aspen chips), *ad lib* access to food (CRM, Expanded, Special Diets Services Witham, United Kingdom), water,carton houses (Rat Corner House, Bio Services B.V., Uden, The Netherlands) and plastic tubes (Rat retreat Amber, Plexx, Elst, The Netherlands) as cage enrichment. The environment was temperature (21±2°C) and humidity (47±3%) controlled with an inversed 12∶12 h light-dark cycle (lights off from 7.00–19.00 hrs) and a radio played constantly as background noise. Animals were handled at least three times a week by the experimenters. All testing procedures occurred in a room different than the animal housing room. During transportation between rooms, exposure to white light was prevented by turning off all lights and covering the cages with a dark sheet.

#### Hot plate test

After an acclimatization period of three weeks, all animals were subjected to the hot plate test. The hot plate apparatus (Model 35100, Ugo Basile, Varese, Italy), was maintained at 50.0±0.1°C. Animals were placed in a glass cylinder of 24 cm diameter on the heated surface of the hot plate and removed immediately when either a hind paw lick or jumping response was observed [24]. The latency until the first of either these responses was scored. A cut-off time of 120 seconds was used to prevent tissue damage.

#### Pavlovian fear conditioning

Three weeks after the hot plate test, 40 animals were subjected to Pavlovian fear conditioning (n = 10 per strain; the remaining 4 animals per strain were used as control animals in cFos analysis). Pavlovian fear conditioning consisted of two sessions. During session 1, the animal was placed on a stainless steel grid (1.2 cm between bars) enclosed by a Plexiglas box of 30×30×25 cm. After an acclimatization time of 20 minutes, the animal was exposed to 10 CS-US pairings. Foot shocks were used as an US, and were elicited by a scrambled electrical pulse of 1 s duration and a stimulus intensity of 0.5 mA, generated with a Grass stimulator (Model S-88, Grass Medical Instruments, Quincy, Mass, USA). The stimuli were delivered to a Grass stimulation isolation unit, a constant current unit controlling the stimulus intensity and a scrambler. Sound stimuli generated by a sound generator (33120 A, Arbitrary Waveform Generator, Hewlett Packard, Palo Alto, CA, USA) were used as CS (10 seconds 2000 Hz tone, 75 dB sound pressure level; M. Fanselow, personal communication, March 3, 2009). The US always coincided with the last second of the CS and the interval between the CS-onsets was 90 seconds.

One day after the first session, the second session started in which the animal was placed in a different Plexiglas box of 39×29×32 cm. After an acclimatization time of 20 minutes, exposure to the CS started (continuous 5 minute 2000 Hz tone, 75 dB sound pressure level). In both sessions, sound stimuli were presented by one speaker mounted on the covering lid of the Plexiglas box and verified by using a Modular Precision Sound Level Meter (type 2231, Brüel & Kjær, Nærum, Denmark). Behaviour was videotaped continuously.

The video recordings were used to score the duration of freezing behaviour, defined as absence of all visible movements with the exception of breathing movements and pendulum motion of the head while the animal sits in a tensed posture [25], during session 1 and 2. Additionally, shock reactivity (also called post shock activity burst), defined as the time between the shock onset and first instance of freezing behaviour [26], was scored during session 1.

#### C-Fos immunohistochemistry and image quantification

Two hours after exposure to the CS in session 2 of Pavlovian fear conditioning, animals were decapitated. The 16 remaining animals (n = 4 per strain) which did not undergo Pavlovian fear conditioning were taken from their home cage and decapitated at the same time point, and served as control animals for baseline c-Fos expression. Subsequently, the brains were removed and frozen in −80°C 2-methyl-butane and stored at −80°C until sectioning. Coronal sections (20 µm) were cut on a cryostat (Leica CM3050S, Leica Microsystems) and mounted on Starfrost adhesive slides (Knittel Glaser, Waldemar Knittel, Germany) and stored at −20°C. For the immunohistochemical detection of c-Fos, rabbit anti-c-Fos (AB-5, Calbiochem, Darmstadt, Germany) was used. During staining the sections were rinsed after every step in 0.01 M phosphate-buffered saline (PBS; pH 7.4). First, the sections were fixed in acetone. Endogenous peroxidase was blocked with H_2_O_2_ (0.1% in PBS) for 30 minutes. Non-specific labeling was blocked with 5% normal donkey serum (NDS) and 1% bovine serum albumin (BSA) in PBS (PBS-BSA 1%+NDS 5%) for 30 minutes before the rabbit anti-c-Fos incubation (1∶4000 in PBS-BSA 1%+NDS 5%, 4°C, ON). Negative controls were incubated with the PBS-BSA 1%+NDS 5% solution. Next, the sections were incubated with the secondary antibody; donkey–anti-rabbit IgG Biotin SP conjugate (1∶400 in PBS-BSA 1%+NDS 5%, Jackson ImmunoResearch Laboratories, Inc., PA, USA) for 45 minutes. Subsequently, the sections were incubated with avidin-horseradish peroxidase solution (1∶250 in PBS-BSA 1%+NDS 5% vectastain ® Elite ABC, Brunswich Chemie, Amsterdam, The Netherlands) for 60 minutes. Diaminobenzidine tetrahydrochloride (DAB, Sigma-Aldrich, St. Louis, MO, USA) solution containing 0.3% ammoniumnickel sulfate (Sigma, A1827) was used to visualize immunoreactivity. Sections were dehydrated with upgraded alcohol concentrations (40%, 80%, 96% and 100%), xylol and subsequently coverslipped. Brain sections images were digitized using an Olympus BX 51 microscope (Olympus, Tokyo, Japan) with a high-resolution digital camera interfaced with a computer. The anatomical localization was aided by use of adjacent Nissl stained sections and illustrations in a stereotaxic atlas [27].

The following subregions of the prefrontal cortex were investigated: the ventral, medial and lateral part of the orbitofrontal cortex (OFC; VO, MO and LO, respectively; +4.20 mm from bregma), cingulated area 1, prelimbic and infralimbic cortex (Cg1, PrL and IL, respectively; +3.00 mm from bregma). Furthermore, subregions of the amygdala en hippocampus were investigated. The amygdala included the basolateral part and central nucleus (BLA and CeN, respectively; −1.72 mm from bregma) and the hippocampus included the CA1 field and the dentate gyrus (CA1 and DG, respectively; −2.92 mm from bregma). In order to identify each region, at least two overt landmarks were used. For quantitative analysis of c-Fos positive cells, the program Leica QWIN (image processing and analysis software, Cambridge, UK) was used. Left and right hemispheres were analysed separately in one section. The number of positive cells was then averaged for each animal and expressed as number per 1.0 mm^2^.

### Experiment 2

#### Animals

Based on the results of experiment 1, two different (i.e. the most contrasting) adult male inbred rat strains (Elevage Janvier, Le Genet St. Isle, France; 8 weeks old at the time of arrival) were selected, including WKY (WKY/KyoRj; n = 11) and FH (Rjlbm: FH; n = 12). Body weights (±SEM) were 226.37 (±2.87) and 193.25 (±5.18) gram at the time of arrival, respectively.

#### Housing conditions

The housing conditions of the animals of experiment 2 were identical to those of experiment 1 (described earlier).

#### Surgery

After an acclimatization period of three weeks, the animals underwent surgery for permanent implantation of epidural electrodes to enable the measurement of SEPs. Anaesthesia was induced in the rat's home cage in a separate room under red light conditions. Anaesthesia of the WKY was induced with 0.25 mg/kg fentanyl (Fentanyl Janssen®, Janssen-Cilag B.V., Tilburg, The Netherlands, 0.05 mg/ml fentanyl citrate) and 0.15 mg/kg dexmedetomidine (Dexdomitor®, Pfizer Animal Health B.V., Capelle a/d IJssel, The Netherlands, 0.5 mg/ml dexmedetomidine hydrochloride). Because of hypertension and haemorrhagic diathesis of the FH [28], [29], dexmedetomidine was replaced with 3 mg/kg midazolam (Midazolam, Actavis B.V., Baarn, The Netherlands, 5 mg/ml midazolam) and 10 mg/kg ketamine (Narketan®10, Vétoquinol S.A., Lure Cedex, France, 115.34 mg/ml ketamine hydrochloride). After loss of the pedal reflex the animal was transported to the surgery room and, after endotracheal intubation, anaesthesia was maintained with isoflurane in 100% O_2_. The animals were provided with 8 ml of saline (s.c.) to support normal fluid balance and eye ointment to prevent drying of the cornea (Ophtosan® oogzalf, ASTfarma B.V., Oudewater, The Netherlands, 10000 IE vitamin A palmitate per gram). Subsequently, the animal was positioned in the stereotactic apparatus (model 963, Ultra Precise Small Animal Stereotaxic, David Kopf Instruments, Tujunga, CA, USA). Body temperature was monitored using a rectal probe thermometer and maintained at 37–38°C with an adjustable electrically heated mattress. In addition to clinical assessment (i.e. pedal reflexes), respiratory rate, heart rate, in- and expired CO_2_ and SpO_2_ were monitored continuously for assessment of anaesthetic depth and anaesthetic drug administration was adjusted appropriately. Following the skin incision but prior to detachment of the periostium from the neurocranium, 3 mg/kg lidocaine solution (Alfacaine 2% plus adrenaline, Alfasan B.V., Woerden, The Netherlands, 20 mg/ml lidocaine hydrochloride and 0.01 mg/ml adrenaline) was applied. Five small wired stainless steel screws (tip diameter 0.6 mm, impedance 300–350 Ω, Fabory DIN 84A–A2, Borstlap B.V., Tilburg, The Netherlands) were implanted epidurally over the vertex (Vx; 4.5 mm caudal to bregma, 1 mm right from midline), primary somatosensory cortex (S1; 2.5 mm caudal to bregma, 2.5 mm right from midline), anterior cingulate cortex (Acc; 1.5 mm rostral to bregma, 0.5 mm lateral from midline) left and right frontal sinus (10 mm rostral to bregma, 1 mm lateral from midline). All electrodes were wired to an 8 pin receptacle (Mecap Preci-Dip 917-93-108-41-005, Preci-Dip Durtal SA, Delémont, Switzerland) and fixed to the skull with antibiotic bone cement (Simplex™ P bone cement with tobramycin, Stryker Nederland B.V., Waardenburg, The Netherlands, 0.5 g tobramycin per 20 g cement powder). The skin was closed in a single layer around the receptacle. Subsequently, anaesthesia was antagonized 0.05 mg/kg buprenorphine (i.p., Buprecare®, AST Farma B.V., Oudewater, The Netherlands, 0.3 mg/ml buprenorphine) and in the case of dexmedetomidine also with 0.6 mg/kg atipamezole (i.p., Antisedan®, Pfizer Animal Health B.V., Capelle a/d IJssel, The Netherlands, 5 mg/ml atipamezole hydrochloride). Anaesthesia was antagonized in the animal's home cage outside the surgery room in a separate room under red light conditions with extra oxygen supplied. After return of purposeful locomotion, the rat was returned to the animals housing room.

Postoperative analgesia was provided with 0.05 mg/kg buprenorphine at 12 hour intervals for 3 days after surgery and 0.2 mg/kg of meloxicam (s.c., Metacam, Boehringer Ingelheim B.V., Alkmaar, The Netherlands, 5 mg/ml) at 24 hour intervals for 2 days after surgery. Animals were weighed daily until the pre-operative weight was reached, and allowed to recover for at least three weeks prior to the start of the first measurement.

#### Hot plate test

The hot plate was performed similar to experiment 1 (as described earlier).

#### Von Frey test

One week after the hot plate test, mechanical nociceptive thresholds were measured. To this end, all animals were subjected to the automated Von Frey test (Dynamic Plantar Aesthesiometer; Ugo Basile Inc, Comerio VA, Italy). The animals were habituated to the testing condition during four days in which they were placed in the Von Frey chamber (26×21×9 cm), positioned on an elevated grid platform, for 30 minutes. After these four days, animals were tested. Before testing, the animals were acclimatized in the Von Frey chamber for 30 minutes. The force applied on the left hind paw started at 0 grams and increased with 5 grams per second, with a maximum of 50 grams. The force at which the paw was withdrawn was recorded automatically. This measurement was repeated five times per animal, with an inter-trial interval of 2 minutes.

#### Somatosensory evoked potential recordings

One week after the Von Frey test, SEPs were recorded. SEPs were evoked by electrical stimuli which were administered as described earlier [17] to the epidermis of the left lateral part of the tail base, using a set of two bar electrodes (brass, diameter 2 mm), tapered towards the contact site and spaced at 3 mm from each other. The electrodes were fixed in a piece of plastic tube which enclosed the tail and was tightened by Velcro for maximal fixation. Stimulation for each SEP consisted of 32 square-waved pulses of a 2 ms duration with a stimulus frequency of 0,5 Hz, generated with a Grass stimulator (Model S-88, Grass Medical Instruments, Quincy, Mass, USA), triggered by dedicated software written in house in a Labview environment (Labview 7.2, National Instruments Netherlands B.V., Woerden, The Netherlands). The stimuli were delivered to a Grass stimulation isolation unit and a constant current unit controlling the stimulus intensity. To prevent the animals from gnawing the cables, an Elizabethan neck collar, developed in house [30], was used during the administration of electrical stimuli. Additionally, the rat's head mounted receptacle was connected to the recording device via a swivel connector (SLC-2, Plastics One, Roanoke, VA, USA). For each SEP recording, the accompanying ipsilateral frontal sinus electrode served as reference and accompanying contralateral frontal sinus electrode served as signal ground. For each SEP, segments of 500 ms, 200 ms pre and 300 ms post stimulus, were recorded and averaged online. All signals were amplified 1 million times, band-pass filtered between 3 and 300 Hz and digitized online at 10 kHz by data acquisition hardware (National Instruments, PCI-6251, Instruments Netherlands B.V., Woerden, The Netherlands). Additionally, a 50 Hz notch-filter was applied to eliminate interference from the power supply system. The SEP measurements were carried out in a Plexiglas box of 40×28×30 cm with a stainless steel electrically grounded bottom, shielded by a Faraday cage. SEPs were recorded in response to stimulus intensities of 0.0, 0.2, 0.5, 1.0, 2.0, 3.0, 4.0 and 5.0 mA given in random order.

## Data and Statistical Analysis

P-values were calculated with SPSS 16.0 (SPSS Inc., Chicago, IL, USA) and q-values [31] were calculated using R software (R Foundation for Statistical Computing, Vienna, Austria, http://www.r-project.org). During all analyses, a backward strategy was adopted in which all non-significant interaction terms were removed. Post hoc tests were performed when appropriate, as noted in the text. Statistical graphics were generated using Sigmaplot 11 (Systat software Inc., Chicago, IL, USA). In the case of repeated measures, a mixed model regression is used. This technique is preferred over the conventional repeated measures ANOVA for several reasons, Firstly, missing values do not result in list-wise deletion of the animal and do not require imputation. Secondly, unlike ANOVA, mixed model regression does not assume sphericity or compound symmetry [32].

### Analyses experiment 1

#### Hot plate test

For the hot plate data a two way ANOVA was used, with strain (FH, BN, WKY and LE), type of response (hind paw lick, jumping) and strain x type of response as independent variables, and latency to response (in seconds) as dependent variable. P-values of <0.05 were considered significant. A Sidak correction was applied in case of post hoc testing. The Q-Q plot of residuals indicated a normal distribution of the dependent variable, and the Levene's test indicated homogeneity of variance. Additionally, a chi-square test was performed to evaluate differences in response types (hind paw lick or jumping) between strains.

#### Pavlovian fear conditioning

During session 1, shock reactivity and freezing behaviour were scored per CS, resulting in 10 repeated measurements. The scorings of the experimenter [MS] correlated highly (Pearson's r = 0.85, n = 80, p<0.001) with the scorings of a second observer who was unaware of the aims and procedures of the experiment. The data was analysed using a mixed model regression, with strain (FH, BN, WKY and LE), CS number (1 to 10) and their interaction as fixed factors. The dependent variables were the duration (in seconds) of freezing behaviour and shock reactivity. A Sidak correction was applied in case of post hoc testing. Grand mean centring was used (i.e. values are centred at 0) for all fixed factors, so the intercept could be interpreted and collinearity was prevented [32]. The best fit for the freezing data was obtained by using the model with a random intercept. The best fit for the shock reactivity was obtained by using the model with a random intercept and random slope for CS number.

For session 2 the duration of freezing behaviour was scored in bins of 30 seconds, resulting in 10 repeated measurements. Additionally, the duration of freezing behaviour during the 30 seconds before the onset of the CS was measured (referred to as “pre-CS freezing”). The scorings of the experimenter [MS] correlated highly (Pearson's r = 0.87, n = 88, p<0.001) with the scorings of a second observer who was unaware of the aims and procedures of the experiment. Two mixed model regressions were carried out: 1) with strain (FH, BN, WKY and LE), pre- and post-CS (pre-CS freezing and the first CS bin) and their interaction as fixed factors (with random intercept as best fit) and 2) with strain (FH, BN, WKY and LE), post-CS-onset bin (1 to 10) and their interaction as fixed factors (with random intercept and random slope for CS bin as best fit). The dependent variable was the duration (in seconds) of freezing behaviour. A Sidak correction was applied in case of post hoc testing. Grand mean centring was used (i.e. values are centred at 0) for all fixed factors, so the intercept could be interpreted and collinearity was prevented [32].

#### C-Fos expression

For the c-Fos determinations two measurements were taken from each individual rat (i.e. left and right hemisphere) for each brain area. For each brain area, the four strains and two groups were analysed using a mixed model regression. The two groups included a control group (i.e. animals which were directly decapitated after they were taken from their home cage) and an experimental group (i.e. animals which were decapitated two hours after exposure to the CS in session 2 of Pavlovian fear conditioning). C-Fos data was analysed using a nested model with a random intercept, with strain and strain x group as fixed factors. This way, the effect of group was analysed for each strain separately. Grand mean centring was used (i.e. values are centred at 0) for all fixed factors, so the intercept could be interpreted and collinearity was prevented [32]. P-values obtained from this analysis were adjusted to maintain a false discovery rate (i.e. type I error) of 5% [31], so that q-values <0.05 were considered significant.

### Analyses experiment 2

#### Hot plate test

For the hot plate data a two way ANOVA was used, with strain (FH and WKY), type of response (hind paw lick, jumping) and strain x type of response as independent variables, and latency to response (in seconds) as dependent variable. P-values of <0.05 were considered significant. The Q-Q plot indicated a normal distribution of the dependent variable, and the Levene's test indicated homogeneity of variance. Additionally, a chi-square test was performed to evaluate differences in response types (hind paw lick or jumping) between strains.

#### Von Frey test

For the Von Frey data the lowest and highest value of the five repeated measurements were deleted, resulting in one average force (in grams) of three repeated measurements per animal as dependent variable, with strain (FH and WKY) as independent variable. The Q-Q plot indicated a normal distribution of the dependent variable. As the Levene's test indicated heteroscedasticity (F = 4.40, p>0.05) the data was analysed using an independent sample t-test not assuming equal variances [33]. A p-value of <0.05 was considered significant.

#### Somatosensory evoked potential recordings

Both amplitudes of positive-to-negative components and latencies of the recorded SEPs were statistically analysed using a mixed model regression. Grand mean centring was used (i.e. values are centred at 0) for all fixed factors, so the intercept could be interpreted and collinearity was prevented [32]. Both for the amplitudes of positive-to-negative components and peak latencies the best fit was obtained by using the model with a random intercept and random slope for stimulus intensity. Dependent variables were the amplitudes of positive-to-negative components, including P1-N1, P2-N2 and P2-N3. P-values ≤0.017 (Sidak correction for the number of dependent variables, namely 3) were considered significant. Fixed factors were recording site (Vx, S1 and Acc), strain (FH and WKY), stimulus intensity (0.2, 0.5, 1.0, 2.0, 3.0, 4.0 and 5.0 mA) and their interactions. In case of significant strain*stimulus intensity interaction simple effects were tested in a Sidak corrected post-hoc test using a nested model, estimating the effect of stimulus intensity per strain (i.e. differences in supramaximal stimulation). Peak latencies of P1, N1, P2, N2 and N3 were analysed similarly as the amplitudes. P-values ≤0.010 (Sidak correction for the number of dependent variables, namely 5). The Q-Q plots of the residuals indicated a normal distribution for all variables.

For the FH no missing values occurred, resulting in the analysis of 84 SEPs per recording site (n = 12 per stimulation intensity). For the WKY, technical problems (i.e. short-circuit) occurred in the S1 - Acc of one rat and the S1 - Vx of one rat, resulting in the analysis of 70 Acc SEPs (n = 10 per stimulus intensity), 63 S1 SEPs (n = 9 per stimulus intensity) and 70 Vx SEPS (n = 10 per stimulus intensity).

## Results

### Results experiment 1

#### Hot plate test

**Figure 2:**
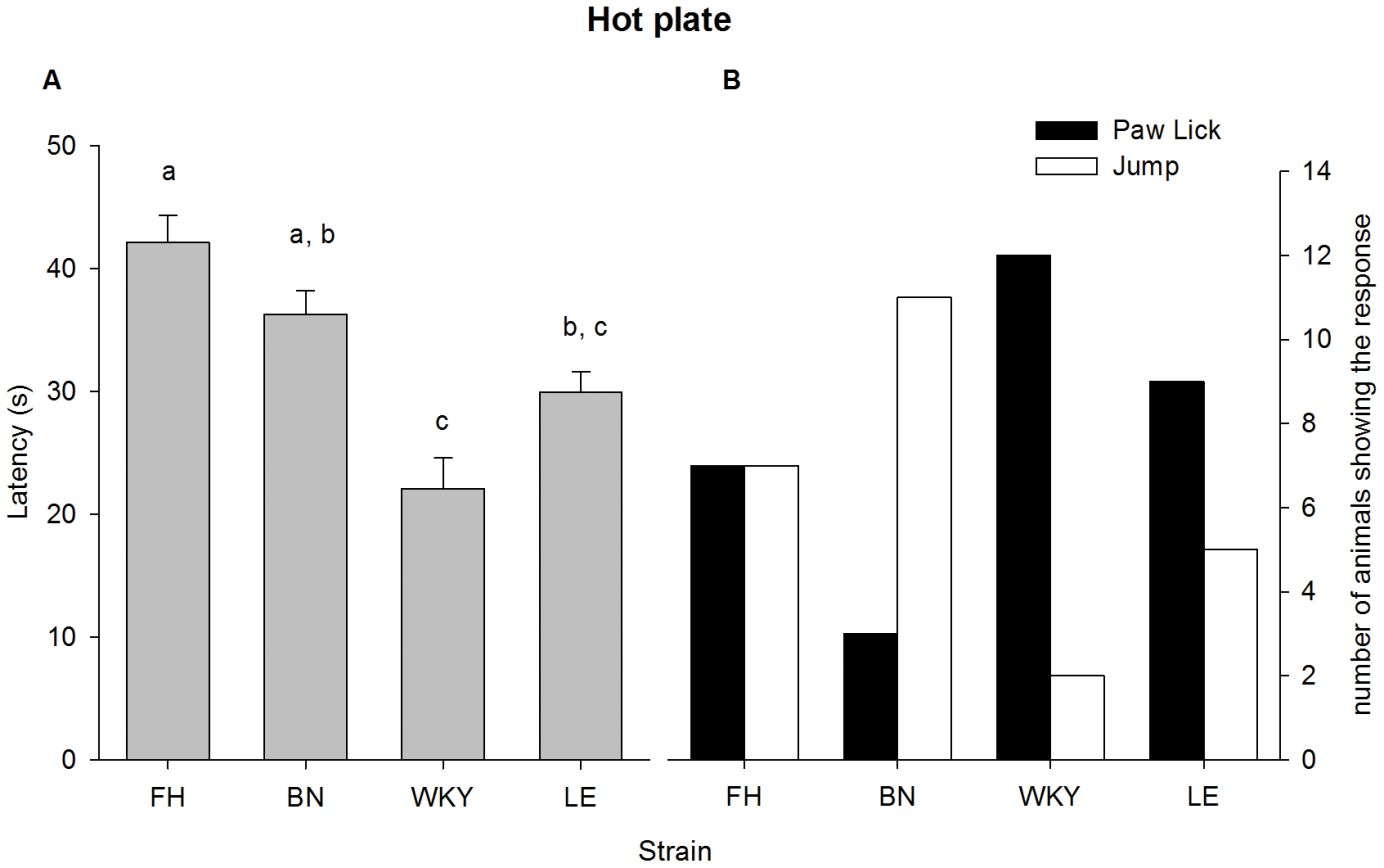
Strain differences in thermal nociception as measured with the hot plate test during the first experiment (see text for details). (A) Latency times differed between strains. Data are represented as mean ±SEM. Values which are different between strains are marked with different characters, i.e. FH = BN, FH>WKY = LE, BN = LE, BN>WKY. (Sidak; p<0.05). (B) Strains differed in their response type, after which they were immediately removed from the hot plate. Data are presented as number of animals showing a response. FH =  Fawn Hooded, BN =  Brown Norway, WKY =  Wistar Kyoto and LE =  Lewis.

None of the rats reached the 120 seconds cut-off time. No effect of type of response was found on latency time (type of response x strain: F_3,48_ = 1.23, p = 0.35; type of response: F_1,51_ = 0.56, p = 0.46). A significant difference was found between strains (F_3,51_ = 15.83, p<0.001), shown in Fig. 2. Post hoc test (Sidak) revealed that the latency time of the FH was significantly longer compared to WKY and LE, the BN showed a significantly longer latency time compared to the WKY.

Furthermore, a significant difference was found between strains in type of response, χ^2^(3, *N* = 56) = 12.36, *p*<0.01, see Fig. 2B.

#### Pavlovian fear conditioning

**Figure 3:**
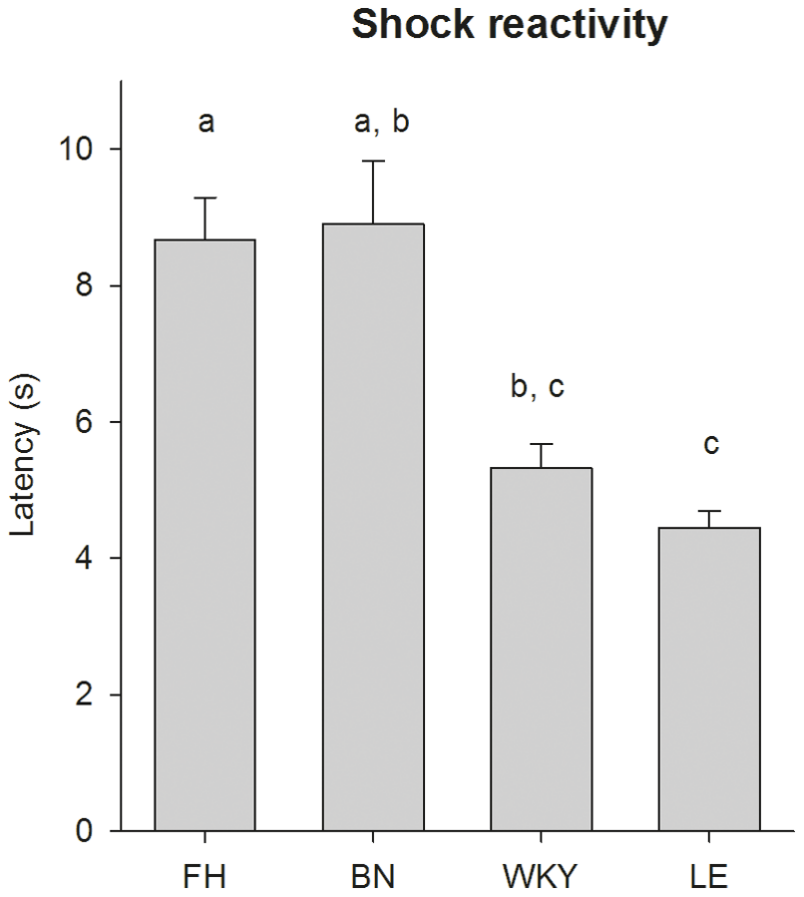
Shock reactivity differed per strain during the first session of Pavlovian fear conditioning. Shock reactivity was defined as “the time between the shock onset and first instance of freezing behaviour” [26]. Data are presented as mean ±SEM. Values which are different are marked with different characters, i.e. FH = BN, FH>WKY = LE, BN = WKY and BN>LE (Sidak; P<0.05). FH =  Fawn Hooded, BN =  Brown Norway, WKY =  Wistar Kyoto and LE =  Lewis.

Shock reactivity during session 1 was not affected by CS number (CS number x strain: F_27,289_ = 1.49, p = 0.059; CS number: F_9,287_ = 1.10, p = 0.36). Shock reactivity did differ significantly between strains (F_3,40_ = 7.58, p<0.001), as shown in Fig. 3. A post hoc test showed that the LE showed significantly less shock reactivity than the FH and BN (Sidak; p<0.05) and the FH showed significantly more shock reactivity than the WKY (Sidak; p<0.01). No significant differences were found between LE and WKY (Sidak; p = 0.92), FH and BN (Sidak; p = 0.83) and WKY and BN (Sidak; p = 0.17).

**Figure 4:**
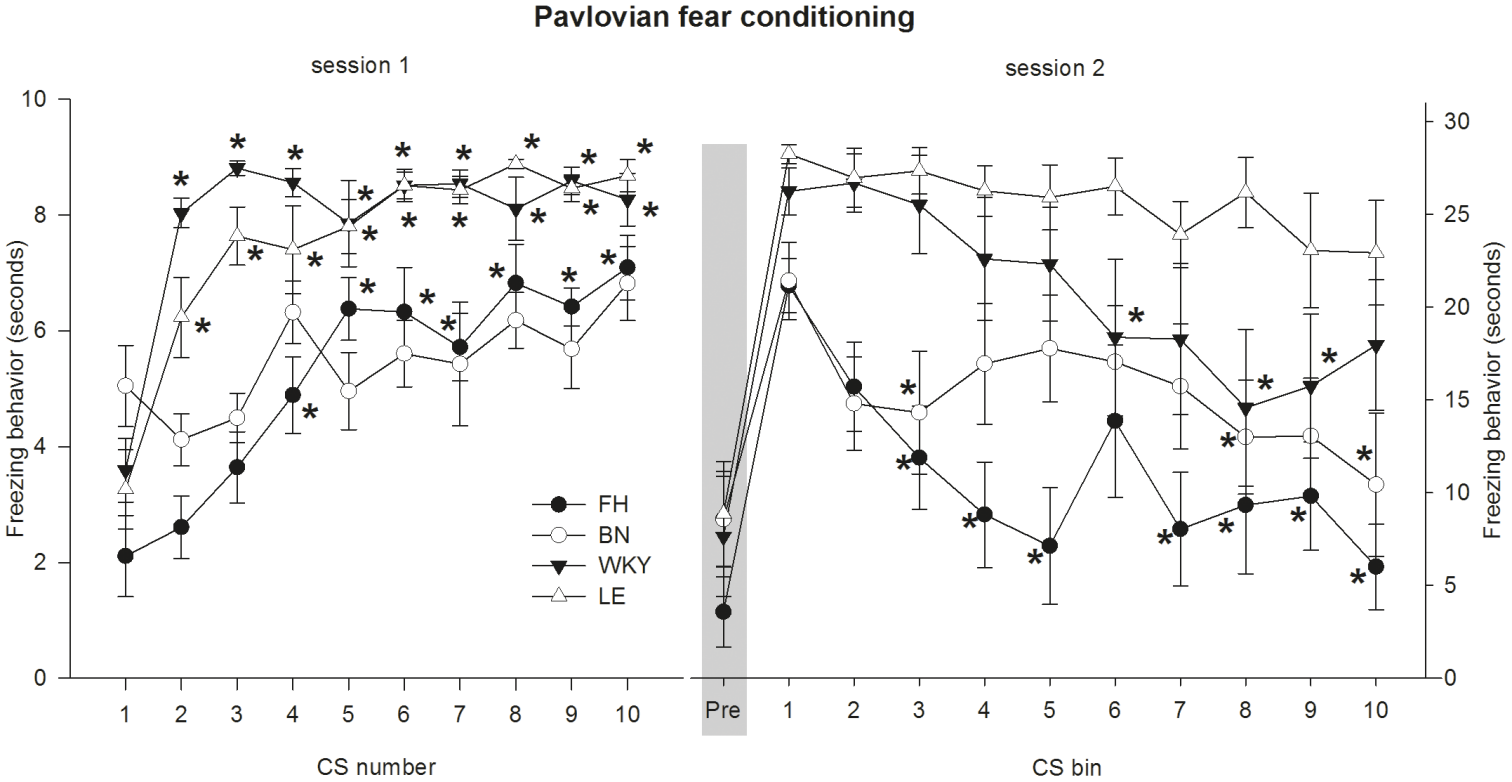
The duration of freezing behaviour during session 1 and 2. During session 1, animals were exposed to 10 CS-US pairings. Foot shocks (0.5 mA, 1 s) were used as an US, and a 10 seconds tone was used as a CS. The FH, LE and WKY showed a significantly increased duration of freezing behaviour over time, whereas the BN did not. During session 2, animals were exposed to a continuous CS of 5 minutes. The duration of freezing behaviour was scored in bins of 30 seconds, resulting in 10 repeated measurements. The FH and BN showed a shorter duration of freezing behaviour than the WKY and LE overall. However, strains significantly differed in duration of freezing behaviour over time.* Sidak p<0.05 compared to the first CS during session 1 and the first CS bin during session 2. Data are presented as mean ±SEM. FH =  Fawn Hooded, BN =  Brown Norway, WKY =  Wistar Kyoto and LE =  Lewis.

In the first session, freezing behaviour was significantly affected by strain (F_3,40_ = 38.81, p<0.001), CS number (F_9,360_ = 27.19, p<0.001) and their interaction (F_27,360_ = 3.67, p<0.001). Overall, the LE and WKY showed a significantly longer duration of freezing behaviour than the FH and BN (Sidak; p<0.001). No significant differences were found between LE and WKY (Sidak; p = 0.84) and FH and BN (Sidak; p = 0.95). In a post hoc analysis (a nested mixed model with CS number and strain x CS number as fixed factors) duration of freezing behaviour during CS numbers 2 to 10 were compared to CS number 1 for each strain. Results are shown in Fig. 4. For the FH, the duration of freezing behaviour was significantly increased during CS number 4 to 10 (Sidak; p<0.001). The WKY and LE both showed a significantly increased duration of freezing behaviour during CS numbers 2 to 10 (Sidak; p<0.001). However, the BN showed no significantly increased duration of freezing behaviour during CS numbers 2 to 10 (Sidak; p>0.05).

In the second session, analysis 1 (i.e. to test for strain differences in duration of freezing behaviour between the pre-CS freezing and the first CS bin) showed that all strains displayed a significantly longer duration of freezing behaviour during the first CS bin compared to freezing before the CS onset (F_1,40_ = 182.32, p<0.001). No difference between strains was found (strain x CS bin: F_3,40_ = 1. 49, p = 0.232; strain: F_3,40_ = 2.61, p = 0.065). Analysis 2 (i.e. to test for strain differences in freezing behaviour over CS bin 1 to 10) showed that during exposure to the CS (i.e. CS bin 1 to 10), freezing behaviour was affected by the CS bin (F_9,261_ = 4.07, p<0.001), strain (F_3,39_ = 10.77, p<0.001) and their interaction (CS bin x strain: F_27,257_ = 1.75, p<0.05). Overall, the LE and WKY showed a significantly longer duration of freezing behaviour than the FH and BN (Sidak; p<0.01). No significant differences were found between LE and WKY (Sidak; p = 0.98) and FH and BN (Sidak; p = 0.85). In a post hoc analysis (a nested mixed model with CS bin and strain x CS bin as fixed factors) the duration of freezing behaviour during CS bin 2 to 10 were compared to CS bin 1 for each strain. Results are shown in Fig. 4.

#### C-Fos expression

**Figure 5:**
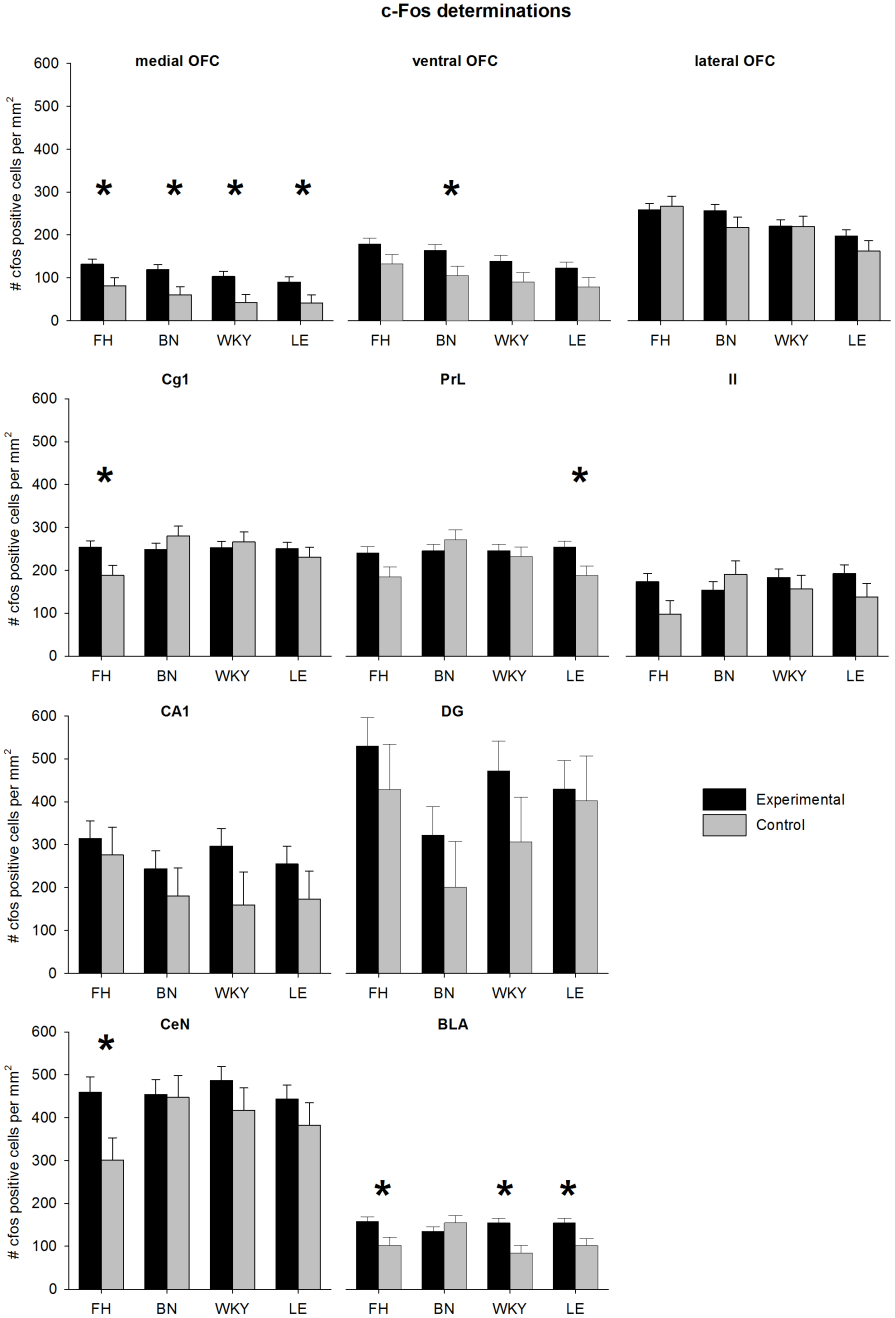
Amount of c-Fos positive cells in different brain areas during conditioned fear after Pavlovian fear conditioning. For each strain the experimental group (n = 10) is compared to control animals of that same strain (n = 4). Data are presented as estimated marginal means ±SEM. * Q-value <0.05 between the experimental and control. FH =  Fawn Hooded, BN =  Brown Norway, WKY =  Wistar Kyoto and LE =  Lewis, OFC =  orbitofrontal cortex, Cg1 =  cingulated area 1, PrL =  prelimbic cortex, IL =  infralimbic cortex, BLA =  basolateral amygdala, CeN central nucleus of the Amygdala, CA1 =  CA1 field hippocampus, DG =  dendate gyrus.

C-Fos expression in the experimental and control groups per strain per brain area are shown in Fig. 5. An effect of group was found in the medial orbitofrontal cortex for all strains (FH: t_56_ = 2.23, q<0.05; BN: t_56_ = 2.63, q<0.05; WKY: t_56_ = 2.69, q<0.05; LE: t_56_ = 2.17, q<0.05), in the basolateral amygdala effects were found for the FH (t_66_ = 2.56, q<0.05), WKY (t_57_ = 3.42, q<0.05) and LE (t_50_ = 2.70, q<0.05). Furthermore, the FH showed significant effects in the central nucleus of the amygdala and cingulated area 1 (t_54_ = 2.53, q<0.05 and t_56_ = 2.40, q<0.05 respectively), the BN showed a significant effect in the ventral orbitofrontal cortex (t_56_ = 2.21, q<0.05), and the LE showed a significant effect in the prelimbic cortex (t_56_ = 2.46, q<0.05).

### Results experiment 2

#### Hot plate test

**Figure 6:**
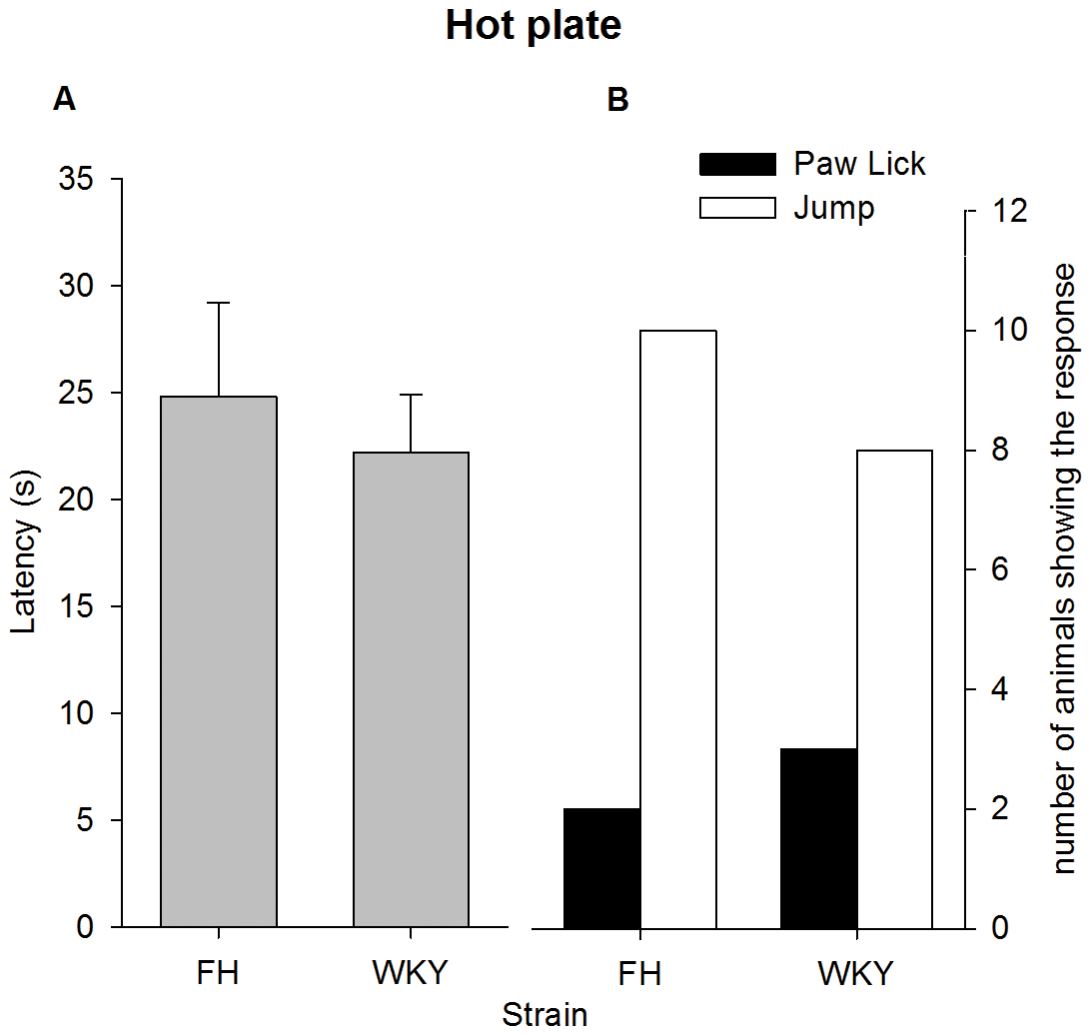
Strain differences in thermal nociception as measured with the hot plate test during the second experiment (see text for details). (A) The type of response did not significantly affect the latency time. Latency times did not differ between strains. Data are presented as mean ±SEM. (B) Strains did not differ in their response type, after which they were immediately removed from the hot plate. Data are presented as number of animals showing a response. FH =  Fawn Hooded, WKY =  Wistar Kyoto.

None of the rats reached the 120 seconds cut-off time. No effect of type of response was found on latency time (type of response x strain: F_1,19_ = 0.09, p = 0.39; type of response: F_1,20_ = 0.04, p = 0.85). No difference was found between strains (F_1,20_ = 2.9, p = 0.10), shown in Fig. 6A. Furthermore, no difference was found between strains regarding type of response, χ^2^(1, *N* = 23) = 0.38, *p* = 0.54, see Fig. 6B.

#### Von Frey test

A significant difference in mechanical thresholds was found between strains (t_15.3_ = 2.26, p<0.05), with the WKY showing a lower threshold compared to FH (mean  = 22.98, SEM = 3.84 and mean  = 30.47; SEM = 2.04 grams, respectively).

#### Somatosensory evoked potential recordings

**Figure 7:**
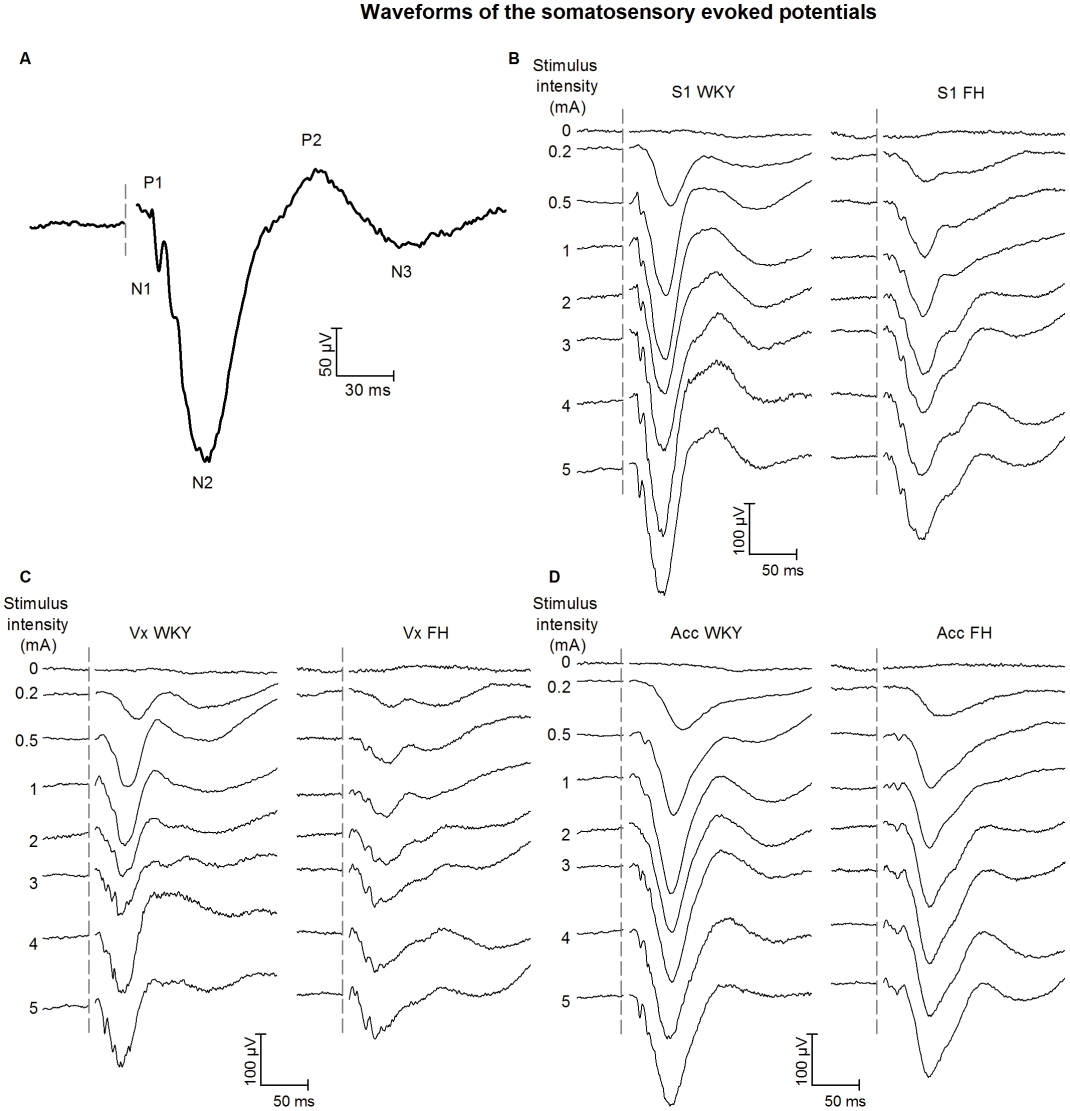
Waveforms of the somatosensory evoked potentials (SEPs) in different rat strains. (A) The S1-SEP waveform and its peak definitions of the WKY at a stimulation intensity of 3 mA. SEP waveforms of the S1 (B, WKY n = 9 and FH n = 12), Vx (C, WKY n = 10 and FH n = 12) and Acc (D, WKY n = 10 and FH n = 12) are shown for both the WKY and FH at different stimulation intensities. The stimulus frequency was 0.5 Hz. SEP waveforms resulted from averaging 32 responses per animal at each stimulus intensity. The dotted line in the curve interruption denotes the stimulus onset. FH =  Fawn Hooded, WKY =  Wistar Kyoto.

The average SEP waveforms of the FH and WKY during different stimulus intensities with its peak definitions are shown in Fig. 7. The previously reported stimulus intensity x recording site interaction [18], [34] did not differ between strains, as none of the variables showed a significant strain x stimulus intensity x recording site interaction. Significant effects of strain, stimulus intensities and their interaction are described below.

#### Amplitudes

**Figure 8:**
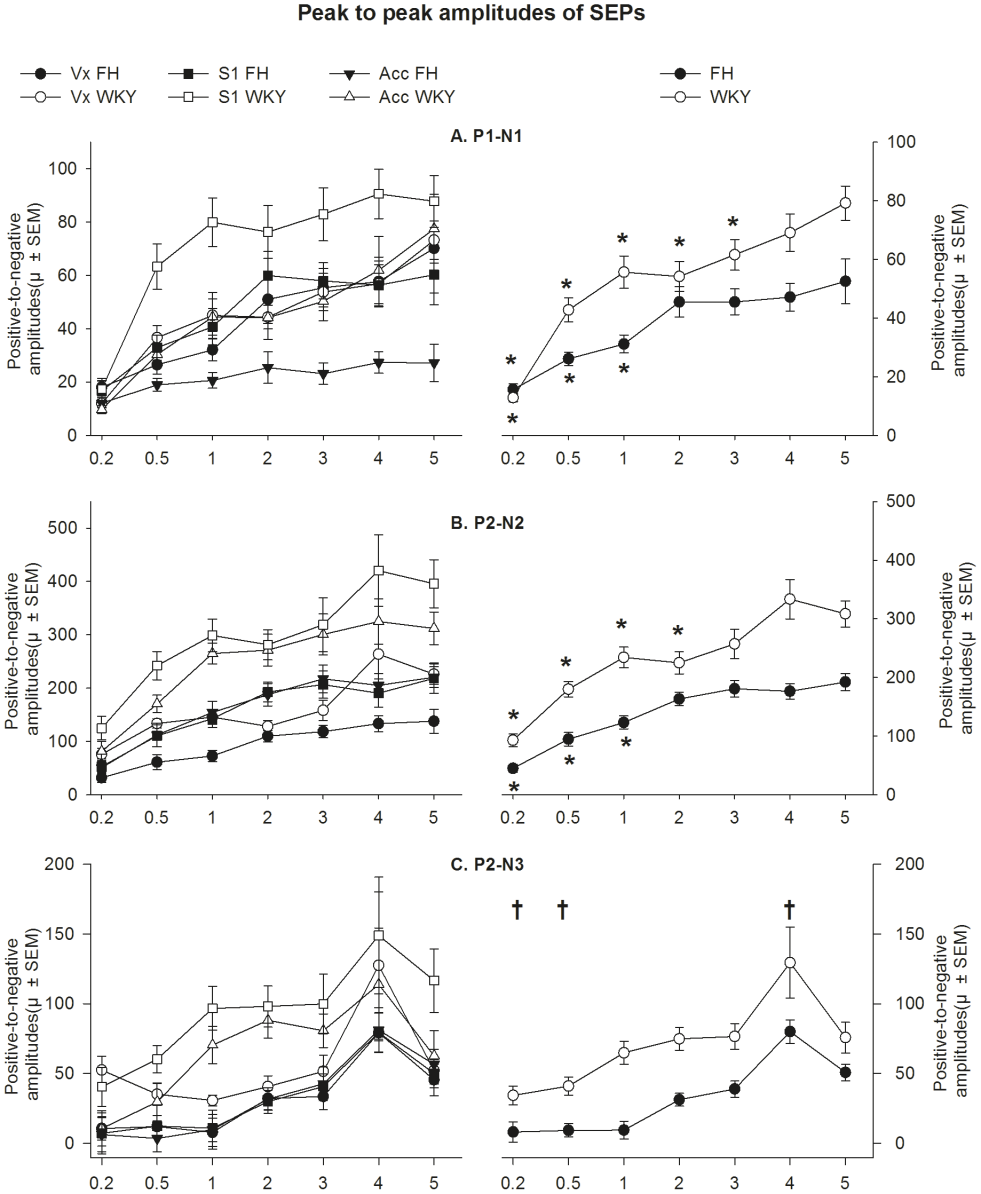
Mean positive-to-negative amplitude per stimulus intensity (x-axis) shown by recording (left) and by strain (right) for the (A) P1-N1, (B) P2-N2 and (C) P2-N3. A different supra-maximal stimulation intensity was found for the WKY and FH for the P1-N1 and P2-N2 (* p<.05 (Sidak) versus 5 mA amplitude within strain). For the P2-N3, a main effect was found for stimulus intensity (**†** p<0.05 (Sidak) versus 5 mA amplitude for both strains). In general, amplitudes were significantly higher in the WKY compared to the FH (see result section for details). Data are presented as µV ±SEM. FH =  Fawn Hooded, WKY =  Wistar Kyoto.

For P1-N1, a strain x stimulus intensity interaction was found (F_6,134_ = 2.77, p<0.017), indicating a different dose-response curve per strain (Fig. 8A). Post-hoc analysis showed that for the FH the P1-N1 amplitudes at 0.2, 0.5 and 1 mA are significantly smaller than at 5 mA (Sidak; p<0.05), whereas P1-N1 amplitudes at 2, 3 and 4 mA did not differ from at 5 mA (Sidak; p>0.05). For the WKY, the P1-N1 amplitudes at 0.2, 0.5, 1, 2 and 3 mA are significantly smaller than at 5 mA (Sidak; p<0.05), whereas 4 mA did not differ from at 5 mA (Sidak; p>0.05). Furthermore, the P1-N1 amplitude was higher for the WKY compared to the FH at 1, 4 and 5 mA (Sidak; p>0.05), whereas no differences were found between strains at 0.2, 0.5, 2 and 3 mA.

For the P2-N2 a strain x stimulus intensity interaction was found (F_6,150_ = 3.52, p<0.01), indicating a different dose-response curve per strain (Fig. 8B). Post-hoc analysis showed that for the FH the P2-N2 amplitudes at 0.2, 0.5 and 1 are significantly smaller than at 5 mA (Sidak; p<0.05), whereas P2-N2 amplitudes at 2, 3 and 4 mA did not differ from at 5 mA (Sidak; p>0.05). For the WKY, the P2-N2 amplitudes at 0.2, 0.5, 1, and 2 mA are significantly smaller than at 5 mA (Sidak; p<0.05), whereas P2-N2 amplitudes at 3 and 4 mA did not differ from at 5 mA (Sidak; p>0.05). Overall, the P2-N2 amplitude was higher for the WKY compared to the FH at 0.5, 1, 2, 3, 4 and 5 mA (Sidak; p>0.05), whereas no differences were found between strains at 0.2 mA.

The P2-N3 amplitude showed no strain x stimulus intensity interaction (F_6,156_ = 1.00, p = 0.43), see Fig. 8C. A main effect was found for both strain (i.e. WKY>FH) and stimulus intensity (F_1,23_ = 34.99, p<0.001 and F_6,157_ = 11.84, p<0.001, respectively). Post-hoc analysis showed that the P2-N3 amplitudes at 0.2 and 0.5 are significantly smaller than at 5 mA (Sidak; p<0.05), P2-N3 amplitudes at 1, 2 and 3 mA did not differ from at 5 mA (Sidak; p>0.05) and P2-N3 amplitude at 4 mA was significantly higher than at 5 mA (Sidak; p<0.05).

#### Latencies

**Figure 9:**
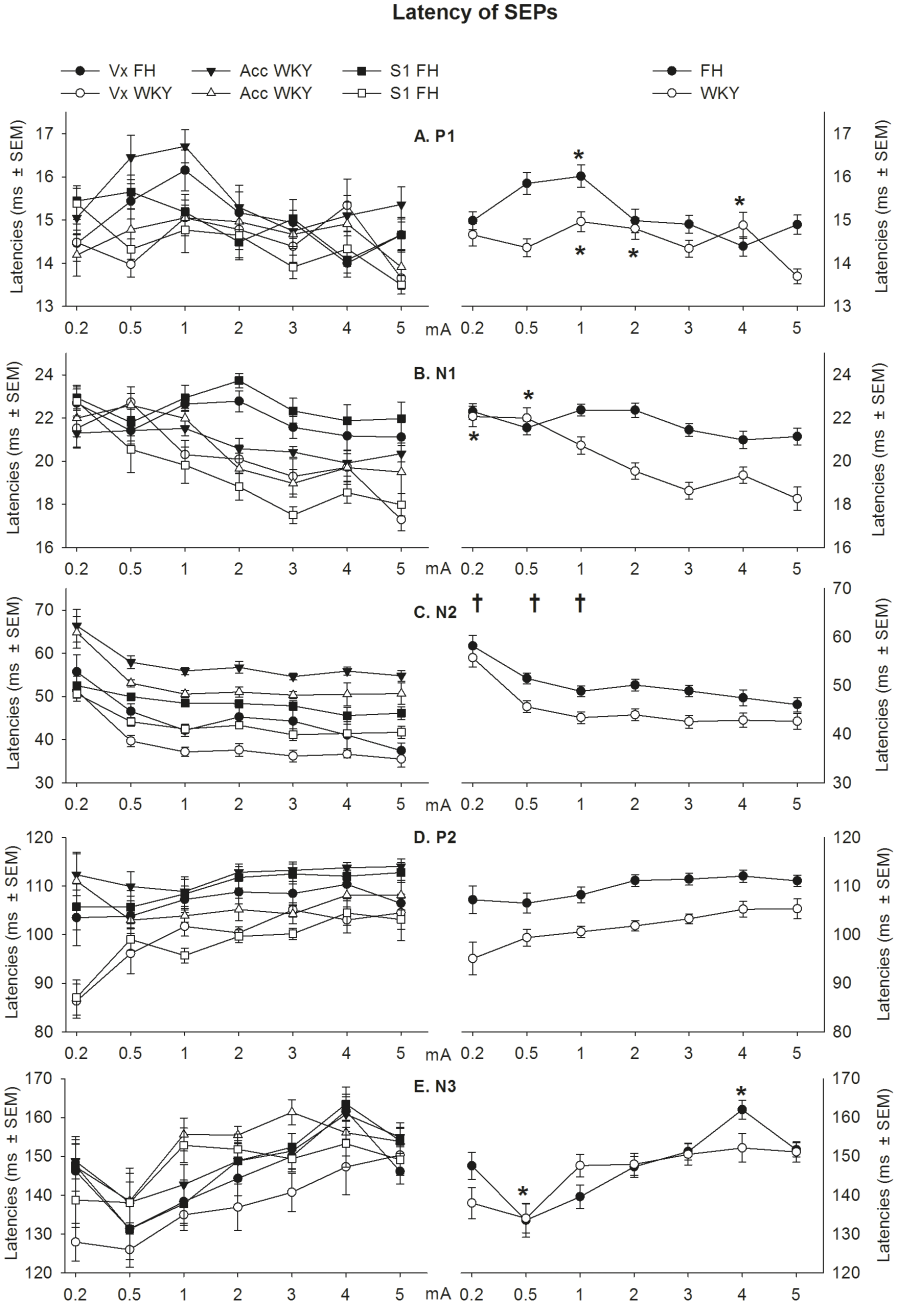
Mean latencies per stimulus intensity (x-axis) shown by strain and recording (left) and by strain (right) for the (A) P1, (B) N1, (C) N2, (D) P2 and (E) N3. A different effect of stimulation intensity was found for WKY and FH for the P1, N1 and N3 (* p<.05 (Sidak) versus 5 mA intensity within strain). For the N3, a main effect was found for stimulus intensity (**†** p<0.05 (Sidak) versus 5 mA intensity for both strains) and strain. For the P2, a main effect of strain was found. Data are presented as ms ±SEM. FH =  Fawn Hooded, WKY =  Wistar Kyoto. See result section for details.

For P1, N1 and N3 a strain x stimulus intensity interaction was found (F_6,169_ = 5.07, p<0.001, F_6,151_ = 4.79, p<0.001 and F_6,133_ = 3.07, p<0.01, respectively), indicating a different dose-response curve per strain for those peaks (see Fig. 9).

For the P1 latencies, post-hoc analysis showed that for the WKY the latencies at 1, 2 and 4 mA are significantly increased compared to at 5 mA (Sidak; p<0.05), whereas latencies at 0.2, 0.5 and 3 mA did not differ from at 5 mA (Sidak; p>0.05). For the FH, the P1 latency of 1 mA was significantly longer compared to at 5 mA (Sidak; p<0.05), whereas 0.2, 0.5, 2, 3 and 4 mA did not differ from at 5 mA (Sidak; p>0.05). Furthermore, the P1 latencies of the FH were significantly longer compared to the WKY at 0.5, 1 and 5 mA (Sidak; p<0.05), whereas no differences were found between strains at 0.2, 2, 3 and 4 mA.

For the N1 latencies, post-hoc analysis showed that for the WKY the latencies at 0.2 and 0.5 mA are significantly longer compared to at 5 mA (Sidak; p<0.05), whereas latencies at 1, 2, 3 and 4 mA did not differ from at 5 mA (Sidak; p>0.05). For the FH, the N1 latencies of different stimulus intensities showed no differences compared to at 5 mA (Sidak; p>0.05). Furthermore, the N1 latencies of the FH were significantly longer compared to the WKY at 3, 4 and 5 mA (Sidak; p<0.05), whereas no differences were found between strains at 0.2, 0.5, 1 and 2 mA.

For the N3 latencies, post-hoc analysis showed that for the WKY the latencies of different stimulus intensities did not differ when compared to at 5 mA (Sidak; p<0.05). For the FH, the N3 latency of 0.5 and 4 mA were significantly shorter and longer compared to at 5 mA, respectively (Sidak; p>0.05), whereas 0.2, 1, 2, and 3 mA did not differ from at 5 mA (Sidak; p>0.05). Furthermore, no differences were found between strains at any stimulus intensity (Sidak; p>0.05).

Both the N2 and P2 did not show a significant strain x stimulus intensity interaction (F_6,153_ = 1.08, p = 0.38 and F_6,157_ = 0.588, p = 0.74, respectively). For the N2 latency both a main effect of strain (F_1,23_ = 63.61, p<0.001) and stimulus intensity (F_6,152_ = 20.07, p<0.001) were found. Latencies shortened with increasing stimulus intensities: the latencies of 0.2, 0.5 and 1 mA were significantly longer compared to at 5 mA (Sidak; p<0.05). Furthermore, the latencies of the FH are longer compared to WKY. For the P2 a main effect of strain (F_1,23_ = 60.02, p<0.001) was found, with the latencies of the FH being longer compared to WKY. No effect stimulus intensity (F_6,157_ = 2.92, p = 0.057) was found.

## Discussion

This is the first study to compare multiple phylogenetically distant inbred rat strains (i.e. FH, BN, WKY and LE) in various tests for pain and nociception. Significant differences in behaviour were found between strains during these tasks, including hot plate, automated Von Frey and Pavlovian fear conditioning. Furthermore, in the two most contrasting strains regarding behaviour during the hot plate test and Pavlovian fear conditioning (i.e. FH and WKY) the SEP was investigated, showing differences in cortical processing of a nociceptive stimulus between these strains. This data therefore shows that the rat strain selected plays a critical role when studying pain and nociception.

### Hot plate test

During experiment 1, thermal nociceptive thresholds differed per strain. The FH and BN showed the highest thermal thresholds, in comparison to the WKY and LE. Strains differed in body weight, which might have influenced latency times. However, a recent study showed that this effect is weak [35] and therefore unlikely to fully account for the large differences in latency in this study. Two types of behavioural endpoints were measured, i.e. the hind-paw lick and the jumping response. Animals were removed from the hot plate immediately after showing one of these behaviours. The type of behavioural endpoint observed, differed per strain: the BN showed mainly the jumping response, while within the WKY strain paw licks were most prevalent. The FH and LE showed both behavioural endpoints with equal prevalence. Therefore, when selecting behavioural endpoints the strain used is a factor to be considered. Since the latency within a strain did not differ between the two types of behavioural endpoints (i.e. paw licking and jumping responses) it is recommended to use both types of behaviours as endpoint for the hot plate test.

During experiment 2 however, the findings of experiment 1 could not be replicated. The FH and WKY did not show differences in latency time nor type of behavioural endpoint. Several factors could explain this difference between experiment 1 and 2. Firstly, unlike the animals in experiment 1, the animals in experiment 2 underwent surgery. Despite adequate pain control and washout period, this procedure could theoretically have influenced outcomes on subsequent tests for pain and nociception. This influence is, however, unlikely to fully explain the lack of strain difference in the hot plate test during experiment 2, as the other tests of pain and nociception in experiment 2 (i.e. Von Frey and SEP) did show a difference in outcome between strains. An alternative explanation involves the fact that the two experiments were carried out in series (i.e. different time periods). Therefore, unrecognized factors could have played a role, such as batch differences or (subtle) unintended environmental differences.

### Pavlovian fear conditioning

The duration of freezing behaviour during session 1 (i.e. the training phase) and 2 (i.e. extinction phase) of Pavlovian fear conditioning differed per strain. During session 1, the WKY, LE and FH showed a learning curve, with the duration of freezing behaviour increasing over the CS-US pairings. Remarkably, the BN did not show a learning curve. However, the finding that shock reactivity of the BN did not differ from the FH and WKY suggests that the US was felt by the BN. The poor performance of the BN in shock motivated tasks has been described previously [36], [37]. However, it has been shown that the BN performs well in other cognitive task [36], suggesting that it does not reflect a general cognitive impairment. During session 2 (i.e. extinction phase) the duration of freezing behaviour over time differed per strain. All strains showed markedly increased duration of freezing behaviour during the first CS bin, compared to the 30 seconds before the CS onset. This suggests that the CS-US association was indeed present in all strains.

The observed differences in the duration of freezing behaviour between the four strains both during session 1 and 2 can represent differences in various underlying domains, including cognition (i.e. learning of the CS-US association [38]), emotional reactivity and coping style (i.e. the response to aversive situations [39], [40]), and pain sensitivity [8], [41], [42]. The exact roles of these domains in documented strain differences in freezing behaviour are yet unclear and need to be addressed in future studies. Importantly, as the degree of freezing duration differs between strains during both the training phase and extinction phase, the rat strain needs to be considered when comparing and performing studies involving (conditioned) freezing behaviour.

### C-Fos expression

Currently, the exact neural substrates involved during expression of conditioned fear, fear memory retrieval and extinction learning are unclear. This study shows that recruitment of brain areas during extinction learning (including fear expression and memory retrieval [43]) after Pavlovian fear conditioning differs per rat strain. This limits the generalizability of study outcomes and comparability of studies investigating this process, especially when different strains or even species are used. Studies investigating the expression of conditioned fear and extinction learning should therefore include multiple rat strains in order to increase the generalizability and comparability of results.

This study is the first to provide evidence that specific brain areas recruited during the expression and retrieval of conditioned fear after Pavlovian fear conditioning differ per rat strain. The influence of specific parts of the procedure on c-Fos expression cannot be determined as shock-controls (i.e. animals undergoing Pavlovian fear conditioning during session 1, but receive no CS during session 2) or CS-controls (i.e. animals receive a CS during session 2, but do not undergo Pavlovian fear conditioning during session 1) were not included. However, this study does show strain differences in recruitment of brain areas induced by the procedure as a whole (i.e. session 1 and 2 of Pavlovian fear conditioning). Although strain differences were present on a behavioural level (i.e. freezing behaviour during session 1 and 2), not all of the brain areas recruited showed differences between rat strains (i.e. the medial OFC was equally recruited in all rat strains). This most likely reflects the presence of general processes during Pavlovian fear conditioning which in itself does differ between rat strains. Some non-significant differences found in this study (see Fig. 5) could be interpreted as trends, unable to reach significance due to a lack of power (cause by relatively few control animals, n = 4 per strain). Therefore, future research should ideally include more control animals. Additionally, future studies should investigate the precise relationship between strain differences and similarities, specific brain areas and specific processes involved in Pavlovian fear conditioning.

### Von Frey test

During the automated Von Frey test the WKY demonstrated a lower mechanical threshold than the FH. In our view, differences in body weight did not influence the automated Von Frey test, as the Von Frey device applied and measured the force administered on a constant surface area on the hind paw. Important to note is that the direction of this strain difference in mechanical threshold is in line with other behavioural pain-related tasks (i.e. hot plate and Pavlovian fear conditioning experiment 1), consistently showing the WKY to react more sensitive to tests of pain and nociception than the FH. The documented difference between strains during the Von Frey test under baseline conditions stresses the importance of strain dependent outcome of baseline measurements in studies of pain and nociception.

### Somatosensory evoked potential recordings

The WKY and FH showed differences in cortical processing of nociceptive stimuli. Regarding the SEP amplitudes, supramaximal stimulation (the stimulation intensity above the intensity yielding maximal SEP amplitudes) was lower for the FH for the P1-N1 and P2-N2 when compared to the WKY, whereas no difference between strains was found for the P2-N3. In general, the amplitudes were higher for the WKY. Especially at higher stimulation intensities, the amplitudes of the WKY continued to increase while the FH showed a maximal response. Altogether, the FH appeared to be less reactive to increasing stimulus intensities when considering both amplitudes and peak latencies (i.e. P1 and N1). Although the functional meaning and underlying mechanism of differences in peak amplitudes and latencies remain to be determined, this research shows that the rat strain should be carefully considered when selecting stimulus intensities upon studying the neural processing of a noxious stimulus using the SEP. The optimal SEP-evoking stimulus intensity and the response to a specific stimulus intensity can differ per rat strain.

### Choice of strain

The choice of the rat strain should be primarily driven by the aim(s) of the research. When a high pain sensitivity is important (for example when studying antinociception), the WKY might be the first choice as this strain showed the highest pain sensitivity during the tests performed within this study. However, when investigating learning curves during conditioning paradigms using noxious stimuli, the FH might be the first choice as this strain shows a gradual learning curve over trials. In conclusion, when selecting a strain for a particular study it should be considered how this strain behaves during the tests used in that study.

Importantly, it should be considered that other factors than rat strain can influence pain behaviours as well, including gender [3], laboratory environment [44], age [45] and circadian rhythm [46]. Furthermore, previous research suggests an interaction between gender and strain differences [3]. Whether laboratory environment, age and circadian rhythm interact with strain differences has not been investigated yet. In order to optimize strain choices in future studies, these factors need to be addressed.

## Conclusions

This is the first study demonstrating that different inbred rat strains show differences in outcomes of tests using noxious stimuli, both on the behavioural and cerebral level. These findings show the significance of the selected rat strain when studying fear conditioning, pain and nociception. In the current literature, reported studies typically include one single strain per study, limiting the generalizability to other species or strains. In order to increase the generalizability of study results, including multiple rat strains is strongly recommended. Additionally, including multiple rat strains in studies of fear conditioning, pain and nociception could contribute to the unravelling of more fundamental underlying mechanisms. Taking strain-dependent differences in performance in tests of nociceptive sensitivity, emotionality, and cognition into consideration is essential in order to facilitate the identification of phenotypes involved in pain and nociception.
